# *G6PD* overexpression protects from oxidative stress and age‐related hearing loss

**DOI:** 10.1111/acel.13275

**Published:** 2020-11-22

**Authors:** Jose M. Bermúdez‐Muñoz, Adelaida M. Celaya, Sara Hijazo‐Pechero, Jing Wang, Manuel Serrano, Isabel Varela‐Nieto

**Affiliations:** ^1^ Institute for Biomedical Research “Alberto Sols” (IIBM) Spanish National Research Council‐Autonomous University of Madrid (CSIC‐UAM Madrid Spain; ^2^ Rare Diseases Networking Biomedical Research Centre (CIBERER) CIBER Carlos III Institute of Health Madrid Spain; ^3^ INSERM ‐ UMR 1051 Institut des Neurosciences de Montpellier Montpellier France; ^4^ Institute for Research in Biomedicine (IRB) Barcelona Spain; ^5^ Hospital La Paz Institute for Health Research (IdiPAZ) Madrid Spain

**Keywords:** aging, ARHL, glutathione, NADPH, TrxR

## Abstract

Aging of the auditory system is associated with the incremental production of reactive oxygen species (ROS) and the accumulation of oxidative damage in macromolecules, which contributes to cellular malfunction, compromises cell viability, and, ultimately, leads to functional decline. Cellular detoxification relies in part on the production of NADPH, which is an important cofactor for major cellular antioxidant systems. NADPH is produced principally by the housekeeping enzyme glucose‐6‐phosphate dehydrogenase (G6PD), which catalyzes the rate‐limiting step in the pentose phosphate pathway. We show here that G6PD transgenic mice (*G6PD*‐Tg), which show enhanced constitutive G6PD activity and NADPH production along life, have lower auditory thresholds than wild‐type mice during aging, together with preserved inner hair cell (IHC) and outer hair cell (OHC), OHC innervation, and a conserved number of synapses per IHC. Gene expression of antioxidant enzymes was higher in 3‐month‐old *G6PD*‐Tg mice than in wild‐type counterparts, whereas the levels of pro‐apoptotic proteins were lower. Consequently, nitration of proteins, mitochondrial damage, and TUNEL^+^ apoptotic cells were all lower in 9‐month‐old *G6PD*‐Tg than in wild‐type counterparts. Unexpectedly, *G6PD* overexpression triggered low‐grade inflammation that was effectively resolved in young mice, as shown by the absence of cochlear cellular damage and macrophage infiltration. Our results lead us to propose that NADPH overproduction from an early stage is an efficient mechanism to maintain the balance between the production of ROS and cellular detoxification power along aging and thus prevents hearing loss progression.

## Graphical abstract

G6PD overexpression contributes to overcome cochlear oxidative damage, prevents hair cell and neuronal loss, and, thereby, delays age‐related hearing loss. Congenital levels of NADPH are increased, thus inducing the expression of antioxidant genes via Nrf2, enhancing antioxidant defense systems, reducing oxidative‐derived damage accumulation, and preventing mitochondrial dysfunction and apoptosis of irreplaceable cochlear cell populations in adult mice.

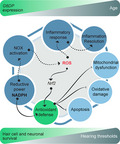

## 1 INTRODUCTION

Age‐related hearing loss (ARHL), or presbycusis, is defined as the progressive and bilateral worsening of auditory function. According to the World Health Organization (2018), one‐third of adults over 65 experience ARHL, which has a strong impact on their quality of life, and is associated with isolation, age‐related cognitive deficit, and neurological disorders. The etiology of ARHL is complex with unresolved genetic components and is strongly influenced by environmental factors such as noise and ototoxic agents, which often aggravate the pathology and contribute to its clinical diversity. Underlying the progression of ARHL is the death of sensory cells and spiral ganglion (SG) neurons in the hearing organ—the cochlea. ARHL initiates in the high‐frequency range and progresses gradually to the low‐frequency range, correlating with the base‐to‐apex degeneration of the cochlea (Gates & Mills, 2005).

Increased reactive oxygen species (ROS) and consequent oxidative stress are a hallmark of aging that contribute to functional decline and to the onset of chronic pathologies (Ferrucci et al., 2019). In this context, the free radical theory of aging postulates that ROS accumulation together with diminished antioxidant defense and impaired oxidative repair triggers mitochondrial dysfunction and causes damage to macromolecules (Balaban et al., 2005). The same scenario occurs in the aged cochlea, with unbalanced inner ear redox homeostasis likely to be the major mechanism underlying hair cell loss (Martinez‐Vega et al., 2015; Wang & Puel, 2018).

Among the cellular antioxidants that play a specific role in ROS homeostasis, thioredoxins (TRXs), glutaredoxins (GRXs), glutathione (GSH), and GSH‐related enzymes constitute the main antioxidant system to prevent or repair oxidative damage to macromolecules (Hanschmann et al., 2013). This system is highly dependent on the reductive power provided by NADPH that is produced by a small number of mammalian cytosolic and mitochondrial enzymes, including isocitrate dehydrogenase 1 and isocitrate dehydrogenase 2 (IDH1 and IDH2), malic enzyme 1 and malic enzyme 3 (ME1 and ME3), glucose‐6‐phosphate dehydrogenase (G6PD), phosphogluconate dehydrogenase (PGD), and glutamate dehydrogenase (GLUD1) (Lewis et al., 2014). Foremost among these is the housekeeping enzyme G6PD, which catalyzes the first and rate‐limiting step of the pentose phosphate pathway and provides pentose phosphates for fatty acid and nucleic acid synthesis (Stanton, 2012). Its importance is underscored by the embryonic lethality in *G6pd* knockout mice (Pandolfi et al., 1995). Interestingly, despite a background of renal oxidative stress (Xu et al., 2010), *G6pd* hypomorphic mice show normal cochlear cytosolic GSH levels and thioredoxin activity, as well as normal hearing up to 5 months of age, the oldest age studied (White et al., 2017). By contrast, loss of *Idh2* increases cochlear oxidative DNA damage and cell death, and accelerates hearing loss progression (White et al., 2018). Using multiphoton fluorescence lifetime imaging microscopy, Majumder et al. (2019) recently showed that a decrease in NADPH levels in hair cells following noise exposure and during aging correlates with oxidative stress and GSH depletion.

*G6PD* transgenic mice (*G6PD*‐Tg) express the human and mouse genes for *G6PD* and present with constitutively increased G6PD activity and NADPH levels. Accordingly, *G6PD*‐Tg mice show reduced levels of oxidative damage and have improved health span during aging (Nobrega‐Pereira et al., 2016). Human and murine G6PD share 93% homology in amino acidic sequence and protein domains, and are considered functionally and structurally equivalent (Notaro et al., 2000; Yang et al., 2013).

Despite the abundant literature addressing the detrimental effects of ROS in the aged cochlea, to our knowledge the potential benefits of NADPH overproduction in the cochlea have not been studied. We show here using *G6PD*‐Tg mice that *G6PD* overexpression efficiently delays ARHL progression by protecting the cochlea from ROS‐derived oxidative damage.

## 2 RESULTS

### 2.1 *G6PD* overexpression in mice delays hearing loss progression

**Figure 1:**
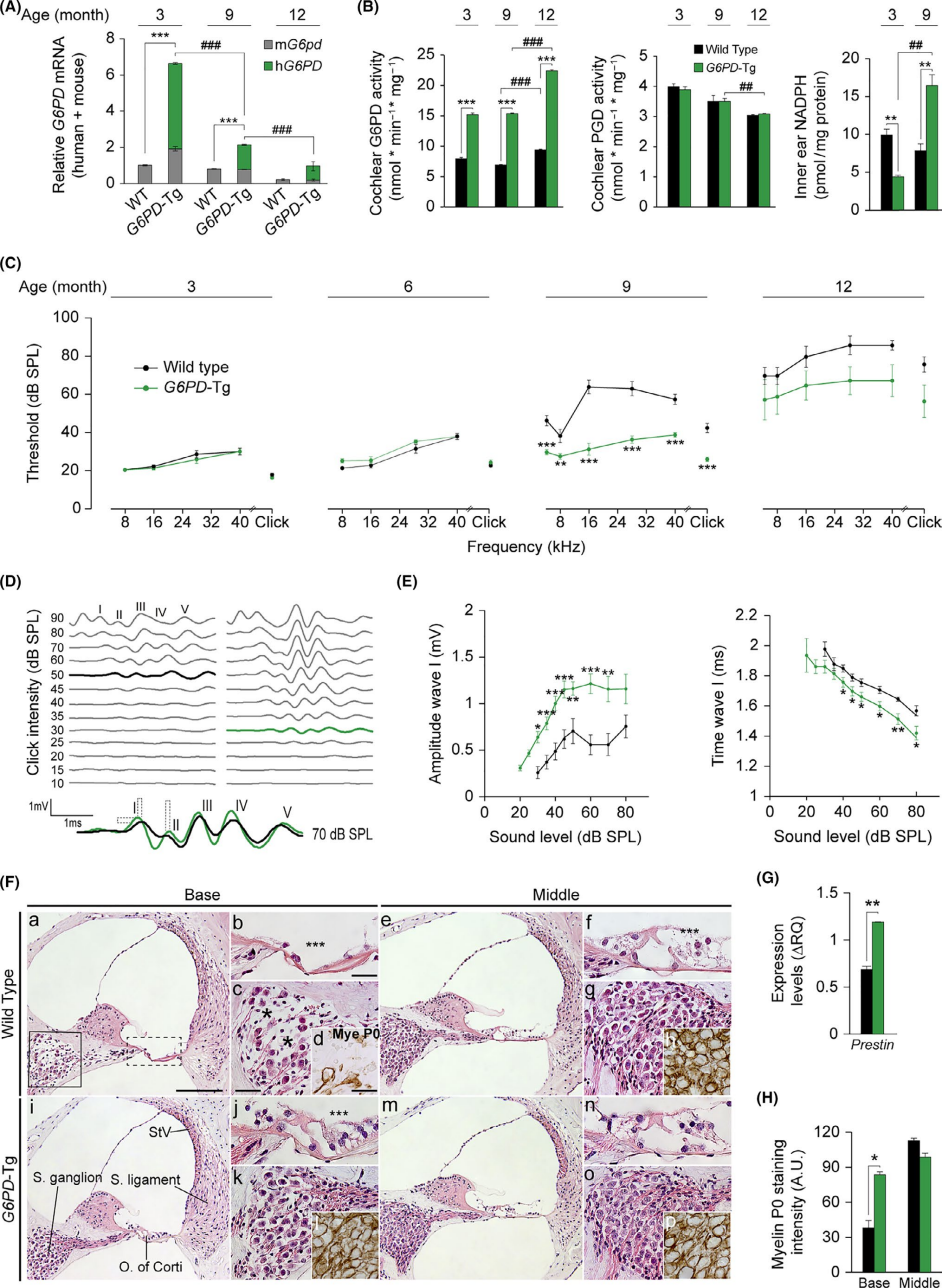
*G6PD*‐Tg mice show better hearing and preserved cochlear cytoarchitecture. (A) Relative levels of total cochlear *G6PD* mRNA in *G6PD*‐Tg and WT mice. Expression levels were calculated as the addition of the 2^−ΔCt^ for the murine and human *G6PD* primers using *18s* as a reference gene and normalization to levels in 3‐month‐old WT mice. RT‐qPCR data are presented as mean ± SEM of triplicate measurements from pooled samples of three 3‐ and 9‐month‐old mice per condition, and mean ± SEM of 3 individual samples of 12‐month‐old mice. (B) G6PD and PGD activities measured from pooled samples of three cochleae in 3‐ and 9‐month‐old mice per condition. NADPH levels measured from pooled inner ears of three 3‐ and 9‐month‐old mice per condition. Data are presented relative to milligram of protein in samples. Data presented as mean ± SEM of at least duplicate measurements. Statistical significance was analyzed by Student's *t* test: *G6PD‐Tg vs WT, # 9‐ and 12‐month‐old mice *vs* 3‐ and 9‐month‐old mice, respectively (*, #*p* < 0.05; **, ##*p* < 0.01; ***, ###*p* < 0.001). (C) ABR thresholds in response to click and tone burst stimuli (4, 8, 16, 28, 40 kHz) in 3‐, 6‐, 9‐, and 12‐month‐old WT and *G6PD*‐Tg mice. (D) Representative ABR recordings in response to click stimulus of WT (left) and *G6PD*‐Tg (right) mice at 9 months of age; thresholds are highlighted. Overlapped ABR waves recorded in response to 70 dB SPL stimuli in WT (black) and *G6PD*‐Tg (green) mice. (E) Input–output ABR wave I amplitude and latency plotted against intensity (dB SPL) for 9‐month‐old mice. Statistical significance was analyzed by Student's *t* test: *G6PD‐Tg vs WT in C and E (*p < 0.05; **p < 0.01; ***p < 0.001). (F) Representative basal and middle turns hematoxylin–eosin staining and MyeP0 immunolabeling of paraffin‐embedded cochlear midmodiolar sections of 9‐month‐old *G6PD*‐Tg and WT mice (*n* = 4 each group). Scale bars: 125 µm in a, 50 µm in b, and c and 25 µm in d. (G) Cochlear gene expression of *Prestin* in 9‐month‐old mice. Expression levels were calculated as ΔRQ normalized to expression data for 3‐month‐old mice for each condition and using *18s* as a reference gene. Values are presented as mean ± SEM of triplicate measurements from pooled samples of 3 mice per condition. (H) MyeP0 intensity of spiral ganglion neurons in 9‐month‐old mice; at least 3 different measurements per turn of each mouse were taken (*n* = 4 mice per group). Values are presented as mean ± SEM. Statistical significance between genotypes was analyzed by Student's *t* test (*p < 0.05; **p < 0.01; ***p < 0.001)

We first confirmed human *G6PD* expression in mouse cochlear samples by RT‐qPCR (Figure 1a). Young *G6PD*‐Tg mice showed a 6.64‐fold increase in total *G6PD* mRNA (endogenous and human) with respect to wild‐type (WT) littermates, with the human transgene contributing more than the mouse *G6pd* transcript to total *G6PD* expression. Moreover, the expression of the *Idh1* and *Pgd* genes for NADPH production was also significantly higher in young transgenic mice than in WT counterparts and decreased gradually along age (Figure S1). Interestingly, endogenous *G6pd* expression decreased unequally in the two genotypes between the ages of 3 and 9 months but not at later time points, while the transgene expression decreased steadily with age. However, total *G6PD* expression levels were higher in *G6PD*‐Tg than in WT mice at all ages studied (Figure 1a). Accordingly, total G6PD activity in the cochlea was higher in *G6PD*‐Tg mice than in WT littermates (Figure 1b), but with a different temporal pattern than that of the transcript, as activity was maintained from months 3 to 9. No differences were observed in cochlear PGD activity between genotypes (Figure 1b), although it decreased with age. We next measured total inner ear, cochlea plus vestibule, and NADPH levels in mice of 3 and 9 months of age. Surprisingly, young *G6PD*‐Tg mice showed lower inner ear levels of NADPH than WT mice, suggesting that *G6PD*‐Tg mice had a higher consumption of NADPH. Conversely, NADPH levels in 9‐month‐old *G6PD*‐Tg inner ears increased significantly compared with both WT mice and young *G6PD*‐Tg mice (Figure 1b).

We next performed a longitudinal analysis of the auditory brainstem response (ABR) in *G6PD*‐Tg and WT mice to follow hearing along age. At 9 months of age, auditory thresholds were significantly lower in *G6PD*‐Tg mice (~25 dB SPL) than in equivalent WT mice, indicating the preservation of hearing over the period studied (Figure 1c). While no significant differences were observed between genotypes at 12 months of age, likely due to the high individual variability, *G6PD*‐Tg mice showed a better response to sound at all frequencies. WT mice showed profound deafness at this age, and therefore, the remainder of studies focused on earlier ages.

The five ABR waves recorded for both genotypes at 9 months of age are shown in Figure 1d. Efficiency of sound conduction was assessed by measuring the amplitude and latency of wave I, which corresponds to the peripheral component of the auditory pathway. No differences in amplitude or latency of wave I were observed between genotypes in young mice (Figure S2B), but significant differences, indicating better hearing, were evident in adult *G6PD*‐Tg mice (Figure 1e). Furthermore, higher amplitudes of ABR waves III and IV were observed in *G6PD*‐Tg mice at this age (Figure S2C).

We next performed histological analyses of cochlear cross sections from young and adult mice, finding no differences in morphology between genotypes in young mice (Figure S2A). By contrast, adult *G6PD*‐Tg mice (Figure 1fi,m) maintained an overall better cochlear cytoarchitecture than WT mice (Figure 1fa,e). Indeed, at the basal turn, the organ of Corti in WT mice showed a flat epithelium, the absence of inner and outer hair cells (IHC and OHC, respectively) (dashed box in Figure 1fa), and also severe neurodegeneration of the SG (solid box in Figure 1fa) (Figure 1fb, c). In addition, no OHC presence could be detected in WT mice at the middle turn (Figure 1ff). Morphological alterations were milder in *G6PD*‐Tg mice, IHCs were not compromised, and missing OHCs were rarer at the basal organ of Corti (Figure 1fj). Moreover, gene expression of the OHC marker *Prestin* was significantly higher in *G6PD*‐Tg mice than in WT mice (Figure 1g). Preservation of myelination in the SG was evaluated by myelin P0 staining (Figure 1fd,h,l,p, quantification in H), and was stronger in the basal SG in *G6PD*‐Tg mice than in WT counterparts, confirming the neuronal preservation observed in the basal turn.

**Figure 2:**
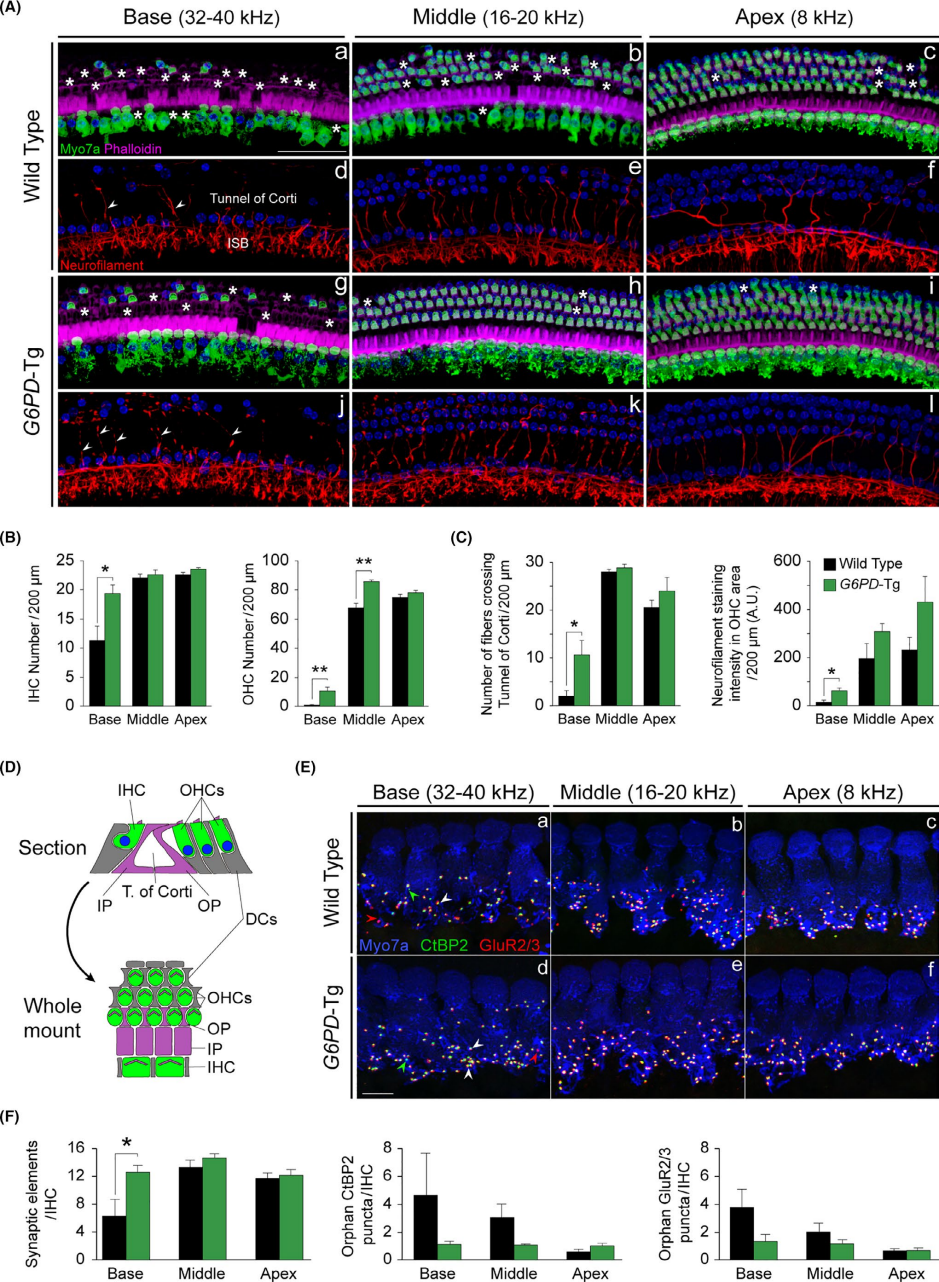
*G6PD*‐Tg mice show preserved hair sensory cells and innervation. (A) Representative confocal images of the organ of Corti basal (32–40 kHz), middle (16–20 kHz), and apical (8 kHz) turns of 9‐month‐old mice of both genotypes immunolabeled for MyoVIIa (green), neurofilament (red), and phalloidin (purple). Asterisks and arrowheads indicate the absence or presence, respectively, of hair cells and fibers. Inner spiral bundle (ISB). Scale bar: 50 µm. (B) Inner hair cell (IHC) and outer hair cell (OHC)counts in basal (32–40 kHz), middle (16–20 kHz), and apical (8 kHz) regions of the organ of Corti (*n* = 3 per condition). (C) Number of fibers that reach the OHC region and neurofilament staining intensity in OHC region (*n* = 3 per condition). (D) Scheme comparing the orientation of the organ of Corti in the midmodiolar section and the whole mount. Hair cells, Deiters’ cells (DCs), and pillar cells (IP & OP) are represented in green, gray, and purple, respectively. (E) Representative confocal images of the organ of Corti basal (32–40 kHz), middle (16–20 kHz), and apical (8 kHz) turns of 9‐month‐old mice of both genotypes immunolabeled for MyoVIIa (blue), CtBP2 (green), and GluR2/3 (red). White arrowheads indicate co‐localized CtBP2‐GluR2/3 puncta, red arrowheads indicate individual postsynaptic marker staining, and green arrowheads indicate individual presynaptic marker staining. Scale bar: 10 µm. (F) Number of synaptic elements (co‐localized CtBP2‐GluR2/3 puncta), orphan CtBP2, and GluR273 puncta per IHC (WT *n* = 3, *G6PD*‐Tg *n* = 4). Data presented as mean ± SEM. Statistical significance between genotypes was analyzed by Student's *t* test (*p < 0.05; **p < 0.01; ***p < 0.001)

We next investigated the cytoarchitecture of the organ of Corti in adult mice using whole mount preparations (Figure 2) and determined hair cell number (myosin VIIa‐positive cells) and organ of Corti innervation (neurofilament fibers) (Figure 2a, quantification in B and C, scheme in D). Organ of Corti degeneration spread toward the apex in both groups, but was more severe in WT than in *G6PD*‐Tg mice (Figure 2aa–f). In line with this observation, IHC and OHC survival was significantly higher in the cochlea of *G6PD*‐Tg mice than of WT mice (Figure 2b). Likewise, when compared with *G6PD*‐Tg mice, innervation of the organ of Corti in the basal region was severely compromised in WT mice, with significantly fewer fibers crossing the tunnel of Corti to innervate the OHC (Figure 2ad–f, j–l and c). We then performed immunolabeling of presynaptic and postsynaptic elements to evaluate afferent synapses in IHC (Figure 2e), finding that the number of co‐localized CtBP2‐GluR2/3 spots per IHC was significantly higher in the basal turn of *G6PD*‐Tg mice than of WT mice (Figure 2f), whereas no significant differences were observed in middle and apical turns.

### 2.2 Antioxidant power is enhanced and oxidative damage is reduced in the cochlea of *G6PD*‐Tg mice

**Figure 3:**
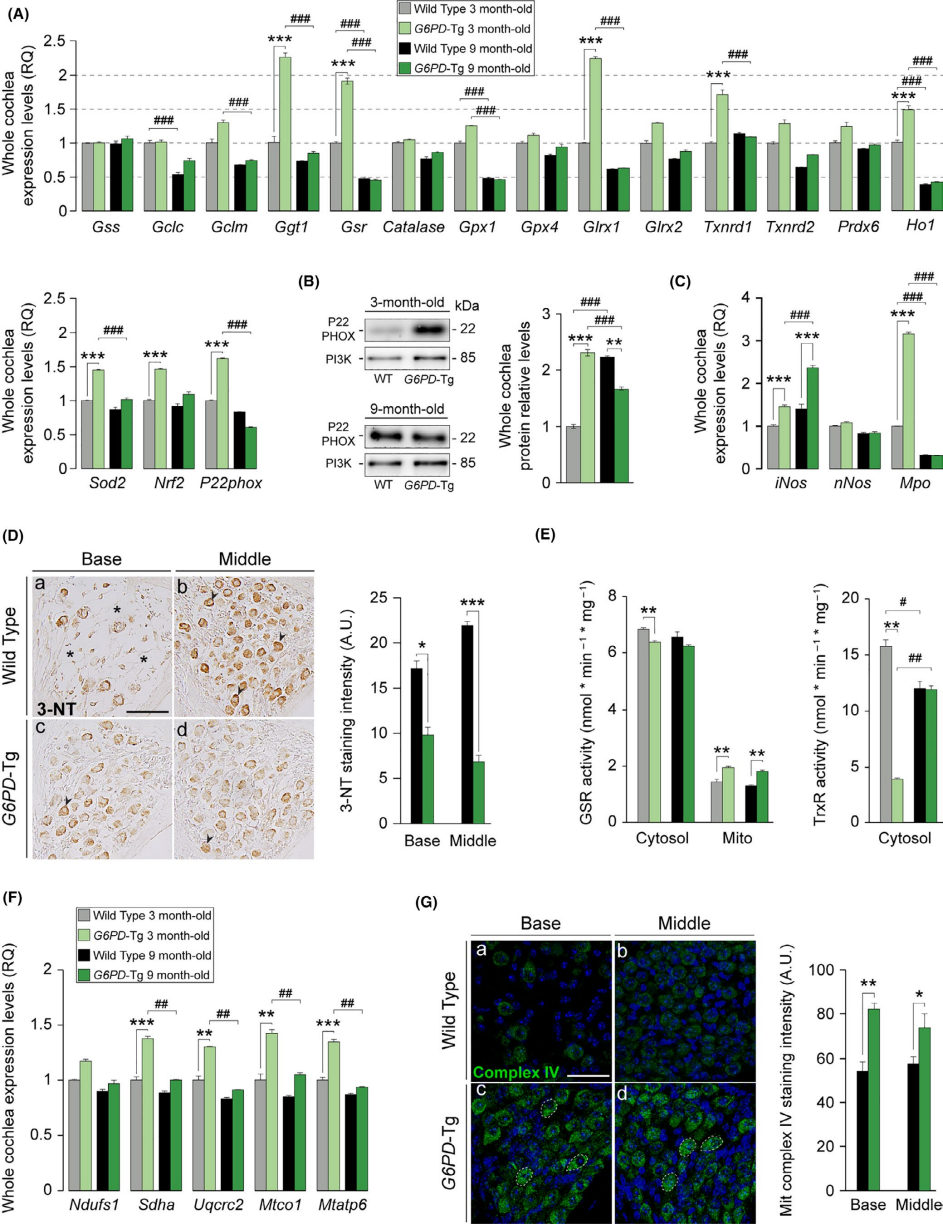
Nine‐month‐old *G6PD*‐Tg mice have reduced oxidative stress, cochlear oxidative damage, and mitochondrial dysfunction. (A) RT‐qPCR gene expression levels of redox enzymes from whole cochleae pooled samples from three 3‐ and 9‐month‐old mice per condition. Expression levels were calculated as 2^−ΔΔCt^ (RQ), using *18s* as a reference gene and normalized to data from 3‐month‐old WT mice. (B) Western blotting of whole cochlear protein extracts, P22PHOX levels were referred to those of PI3K and normalized to data from 3‐month‐old WT mice. Data presented as mean ± SEM of duplicate samples of pooled samples of three 3‐ and 9‐month‐old mice per condition. (C) RT‐qPCR gene expression levels of nitric oxide synthase isoforms and myeloperoxidase from whole cochleae pooled samples from three 3‐ and 9‐month‐old mice per condition. Expression levels were calculated as 2^−ΔΔCt^ (RQ), using *18s* as a reference gene and normalized to data from 3‐month‐old WT mice. Data presented as mean ± SEM of triplicate measurements. Statistical significance between genotypes and stages was analyzed by Student's *t* test: *G6PD‐Tg vs WT, # 9‐month‐old mice vs 3‐month‐old mice (*, #*p* < 0.05; **, ##*p* < 0.01; ***, ###*p* < 0.001). (D) Representative microphotographs of 3‐nitrotyrosine labeling of basal and middle turns of 9‐month‐old *G6PD*‐Tg and WT mice. Asterisks indicate absence of neurons, and arrowheads indicate presence of positive staining. Scale bar: 50 µm. Staining intensity in spiral ganglion (SG) neurons from mice; at least 3 different microphotographs per turn were evaluated (*n* = 3 mice per experimental group). Values presented as mean ± SEM. (E) GSR and TrxR activity in cytosolic and mitochondrial (only GSR) fractions measured from pooled samples of three cochleae from three 3‐ and 9‐month‐old mice per condition. Values presented relative to milligram of protein. Data presented as mean ± SEM of at least duplicate measurements. Statistical significance between genotypes and stages was analyzed by Student's *t* test: *G6PD‐Tg vs WT, # 9‐month‐old mice vs 3‐month‐old mice (*, #*p* < 0.05; **, ##*p* < 0.01; ***, ###*p* < 0.001). (F) RT‐qPCR gene expression levels of mitochondrial complexes components from whole cochleae pooled samples from three 3‐ and 9‐month‐old mice per condition. Expression levels were calculated as 2^−ΔΔCt^ (RQ), using *18s* as a reference gene and normalized to data from 3‐month‐old WT mice. Data presented as mean ± SEM of triplicate measurements. Statistical significance between genotypes and stages was analyzed by Student's *t* test: *G6PD‐Tg vs WT, # 9‐month‐old mice vs 3‐month‐old mice (*, #*p* < 0.05; **, ##*p* < 0.01; ***, ###*p* < 0.001). (G) Mitochondrial complex IV staining intensity of positive stained area of the SG of 9‐month‐old mice. At least 3 different measurements per turn were taken (*n* = 4 WT, *n* = 3 *G6PD*‐Tg). Scale bar: 50 µm. Values are presented as mean ± SEM. Statistical significance between genotypes was analyzed by Student's *t* test (*p < 0.05; **p < 0.01; ***p < 0.001)

To analyze the impact of NADPH overproduction for the cochlear antioxidant system, we surveyed the expression of genes involved in antioxidant defense in mice at 3 and 9 months of age (Figure 3a). *G6PD*‐Tg and WT cochleae showed similar expression levels of genes encoding the GSH synthesis enzyme glutathione synthetase (*Gss*) and the glutamate–cysteine ligase catalytic and regulatory subunits *(Gclc* and *Gclm*) at both stages. By contrast, the expression of the GSH recycling enzyme γ‐glutamyl transferase 1 (*Ggt1*) was significantly higher in young *G6PD*‐Tg mice than in equivalent WT mice. Similarly, the transcription levels of major antioxidant genes, including superoxide dismutase 2 (*Sod2*) and hemoxygenase 1 (*Ho1*), as well as those encoding enzymes dependent on NADPH for their reductive reactions, such as glutathione reductase (*Gsr*), glutaredoxin 1 (*Glrx1*), and thioredoxin reductase (*Txnrd1*) (Scheme in Figure S3), were higher in the cochlea of young transgenic mice than in equivalent WT mice. The expression of transcription nuclear factor erythroid 2‐related factor 2 (*Nrf2)*, a master regulator of several antioxidant genes, was also higher in the cochleae of *G6PD*‐Tg mice than of WT mice (Figure 3a), but only in young mice. Of note, gene (Figure 3a) and protein (Figure 3b) expression of the superoxide‐generating NADPH oxidase (NOX) essential component *P22phox* was also significantly higher in young but not adult *G6PD*‐Tg mice. Indeed, protein levels decreased significantly in adult *G6PD*‐Tg mice as compared with WT counterparts. The superoxide anion can react with nitric oxide generated by the action of nitric oxide (NO) synthases to produce RNS (nitrogen reactive species). This family of antimicrobial molecules can trigger nitrosative stress and cellular damage by forming peroxynitrite from superoxide and nitric oxide. Likewise, myeloperoxidase (MPO) is an antimicrobial enzyme that consumes hydrogen peroxide and produces hypochlorous acid to fight pathogens. Transcripts for *Mpo* and nitric oxide synthase inducible isoform (*iNos*), but not the neuronal nitric oxide synthase isoform (*nNos)*, were significantly higher in the cochlea from *G6PD*‐Tg mice than from WT mice at 3 months (*Mpo* and *iNOS*) and at 9 months (*iNOS*) (Figure 3c).

Overall, these data indicate that there is an early induction of antioxidant defense enzymes in young transgenic mice that contribute to prevent and repair accumulated oxidative damage in the cochlea, between 3 and 9 months of age. Nonetheless, the observed increase in P22PHOX protein levels and NO production could increase peroxynitrite formation, leading to the oxidative modification of protein residues, such as tyrosine nitration. We thus measured tyrosine nitration by 3‐nitro‐L‐tyrosine (3‐NT) staining in 9‐month‐old animals (Figure 3d), finding lower staining intensity in basal and middle turn SG neurons in *G6PD*‐Tg mice, suggesting the presence of more robust antioxidant machinery in these animals. Consequently, we measured GSR and TrxR activities in cytosol and mitochondrial cochlear fractions. Both enzymatic activities in the cytosolic fraction were significantly lower in young *G6PD*‐Tg mice than in WT littermates and were equivalent in 9‐month‐old animals (Figure 3e). Also, TrxR activity increased significantly from 3 to 9 months of age in *G6PD*‐Tg mice. Whereas mitochondrial TrxR activity, corresponding to the *Txnrd2* isoform, was undetectable in mitochondrial extracts, GSR activity in mitochondrial fractions was significantly higher in *G6PD*‐Tg than in WT cochleae at both stages. As mitochondrial GSR was the predominant isoform, we evaluated mitochondrial integrity by measuring the transcript levels of some major components of the mitochondrial respiratory chain complexes (Figure 3f). mRNA levels of *Sdha* (complex II), *Uqcrc2* (complex III), *Mtco1* (complex IV), and *Mtap6* (complex V) were higher in young *G6PD*‐Tg mice than in WT mice. These data suggest a difference in mitochondrial mass and function in the cochlea at this young age. No differences in expression were observed in adult mice at the age of 9 months; however, there was a higher intensity of mitochondrial complex IV (cytochrome C oxidase) immunolabeling in 9‐month‐old *G6PD*‐Tg SG neurons in basal and middle cochlear turns (Figure 3g).

### 2.3 *G6PD* overexpression influences mitochondrial biogenesis and stimulates the inflammatory response

**Figure 4:**
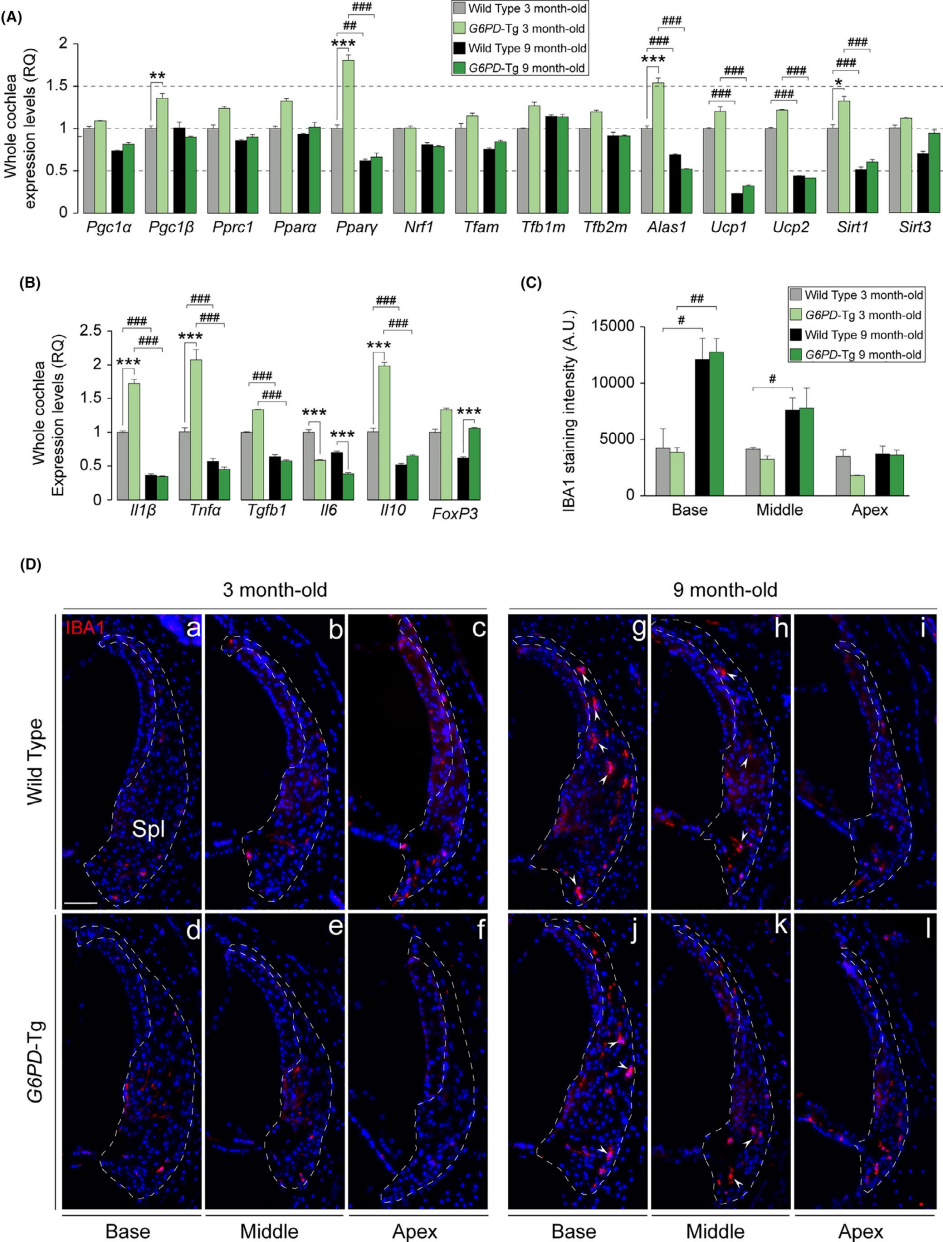
Inflammatory response and macrophage infiltration in the cochlea. (a and b) RT‐qPCR gene expression levels of mitochondrial biogenesis program genes and pro‐ and anti‐inflammatory cytokines from 3 cochleae pools per condition of 3‐ and 9‐month‐old mice, respectively. Expression levels were calculated as 2^−ΔΔCt^ (RQ), using *18s* as a reference gene and normalized to WT data. Data presented as mean ± SEM of triplicate samples. (C) IBA1 staining intensity of the spiral ligament (*n* = 3 WT, *n* = 3 *G6PD*‐Tg). Values are presented as mean ± SEM. Statistical significance was analyzed by Student's *t* test: *G6PD‐Tg vs WT, # 9‐month‐old mice vs 3‐month‐old mice (*, #*p* < 0.05; **, ##*p* < 0.01; ***, ###*p* < 0.001). (D) Representative cochlear cross‐cryosections immunolabeled for IBA1 showing the spiral ligament (Spl) of the basal, middle, and apical turns (outlined) of both genotypes of 3 (a–f) and 9‐month‐old (g–l) mice. Arrowheads highlight positive staining of macrophages. Scale bar: 25 µm

Age‐related mitochondrial dysfunction is tightly linked to ROS production and is characterized by a loss of efficiency in the electron transport chain that leads to energy depletion. Our data thus far point to a better preservation of mitochondrial function in *G6PD*‐Tg mice. To further explore whether *G6PD* overexpression promotes mitochondrial biogenesis, we measured the mRNA levels of the peroxisome proliferator‐activated receptor γ coactivator 1 family members (*Pgc1α*, *Pgc1β*, and *Pprc*) in addition to PGC‐1‐dependent genes (Figure 4a). We found that the expression of the coactivator *Pgc1β* and the nuclear receptor *Pparγ* (peroxisome proliferator‐activated receptor γ) was higher in young *G6PD*‐Tg mice than in WT littermates. By contrast, no differences were observed in the expression of downstream targets *Nrf1*, *Tfam*, *Tfb1m*, or *Tfb2m*. The expression of *Pgc1a*, the *Pgc1β* target gene *Alas1* (δ‐aminolevulinate synthase 1), and *Sirt1*, which induces *Pgc1a* expression, was also upregulated at this age. Finally, the expression levels of the mitochondrial uncoupling proteins 1 and 2 (*Ucp1* and *Ucp2*), which are PGC‐1 downstream targets responsible for energy homeostasis by uncoupling respiration from ATP production, were reduced significantly in both genotypes at 9 months of age.

Up‐regulation of the pro‐inflammatory response is another key factor for aging progression. As shown in Figure 3c, we detected an increase in the transcript levels of *P22phox*, *Sod2*, *iNos*, and *Mpo* in *G6PD*‐Tg mice, suggesting that *G6PD* overexpression could be a trigger of the inflammatory response *via* superoxide and nitric oxide production. Macrophage infiltration is associated with cytokine production and increased phagocytic activity, particularly in the lateral wall during aging. To examine this, we studied the gene expression of several pro‐ and anti‐inflammatory cytokines and performed immunostaining for the pan‐macrophage marker IBA1 (ionized calcium‐binding adapter molecule 1) in the cochlea of 3‐ and 9‐month‐old *G6PD*‐Tg and WT mice. We found that levels of pro‐inflammatory *Il1ß* and *Tnfα* and anti‐inflammatory *Il10* were significantly higher in transgenic mice than in WT mice, whereas the expression of *Il6* was significantly lower, and this was maintained at 9 months of age (Figure 4b). Notably, in addition to *Il10*, the expression of *FoxP3*, a transcriptional repressor of cytokine promoters, was higher in *G6PD*‐Tg mice than in WT mice, suggesting that the early inflammatory response observed was resolved. Indeed, no differences were detected in macrophage infiltration between genotypes, as assessed by IBA immunolabeling. IBA1 intensity exhibited a base‐apex gradient in both genotypes in adult mice, which correlated with the observed temporal pattern of hearing loss (Figure 4c,d).

### 2.4 Pro‐apoptotic signaling and apoptosis is decreased in *G6PD*‐Tg cochlea

**Figure 5:**
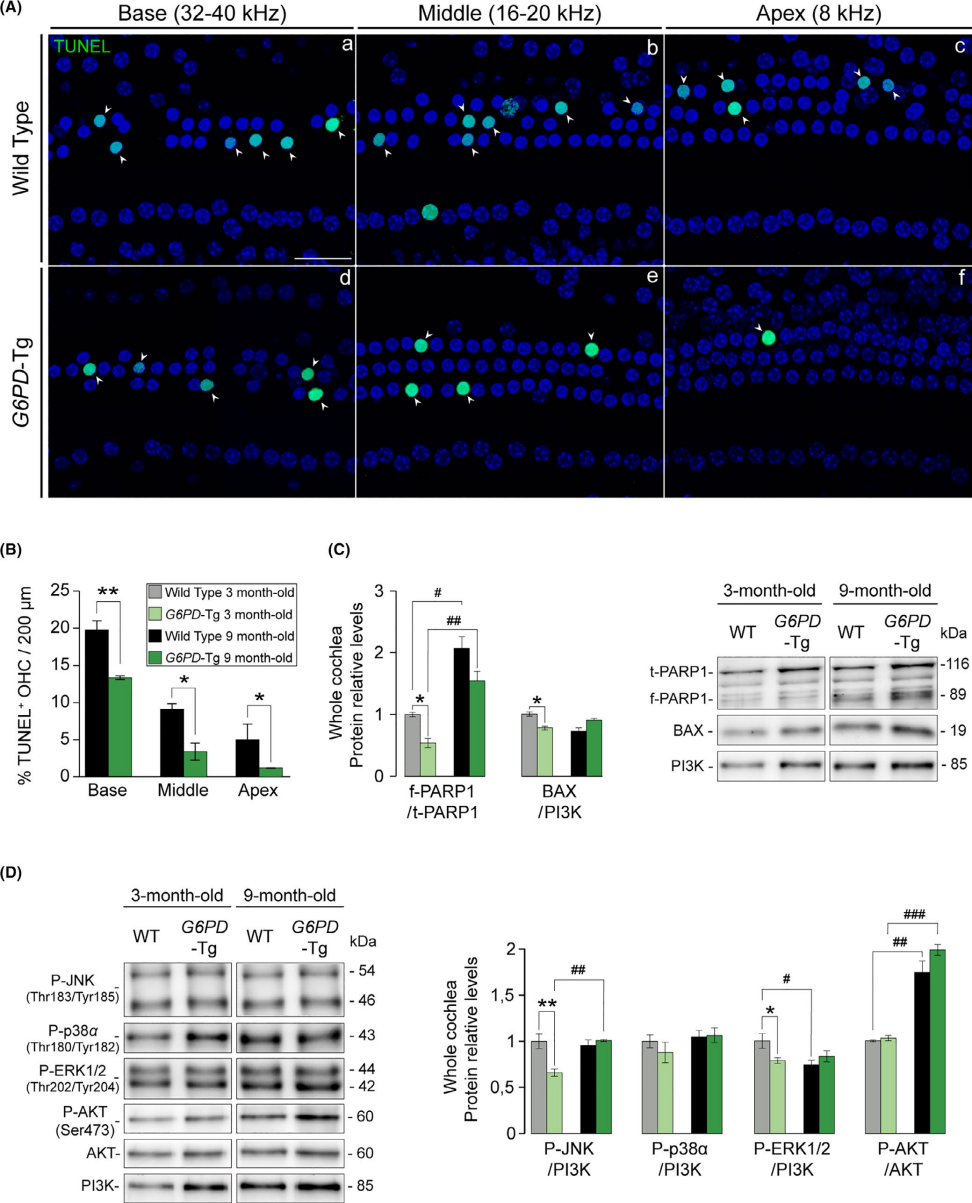
Diminished apoptotic cell death in 9‐month‐old *G6PD*‐Tg mice. (A) TUNEL assay of organ of Corti whole mounts of 9‐month‐old mice. Representative confocal images of basal (32–40 kHz), middle (16–20 kHz), and apical (8 kHz) regions of the organ of Corti are shown. Arrowheads indicate TUNEL^+^ staining. Scale bar: 25 µm. (B) Percentage of TUNEL^+^ outer hair cells (OHC) in 200‐µm sections of basal (32–40 kHz), middle (16–20 kHz), and apical (8 kHz) regions of the organ of Corti (*n* = 3 per condition). Statistical significance between genotypes was analyzed by Student's *t* test (*p < 0.05; **p < 0.01; ***p < 0.001). (c and d) Cochlear protein levels were analyzed by Western blotting of pooled samples from three 3‐ and 9‐month‐old mice per condition. Representative blots and quantifications are shown for pro‐apoptotic and pro‐survival proteins. Protein levels were calculated as a ratio f‐PARP1/t‐PARP1, P‐AKT/AKT, or using PI3K as loading controls, and then normalized to data from 3‐month‐old WT mice. Values presented as mean ± SEM. Statistical significance between genotypes was analyzed Student's *t* test: *G6PD‐Tg vs WT, # 9‐month‐old mice vs 3‐month‐old mice (*, #*p* < 0.05; **, ##*p* < 0.01; ***, ###*p* < 0.001)

Lastly, we studied possible differences in the cell death machinery in the cochlea of *G6PD*‐Tg and WT mice. Loss of OHC by apoptosis measured by TUNEL staining was confirmed in whole mounts of the organ of Corti prepared from basal, middle, and apical turns of 9‐month‐old cochleae (Figure 5a), with a significantly lower number of TUNEL‐positive cells in all the cochlear turns in *G6PD*‐Tg mice than in WT mice (Figure 5b). This correlated well with lower cleaved PARP1/total PARP1 ratios, BAX levels, and stress kinase JNK activation in cochlear protein extracts of young *G6PD*‐Tg mice (Figure 5c,d). In addition, ERK1/2 activation was lower in young but not in adult transgenic cochlea, whereas the activation of pro‐survival AKT was increased in 9‐month‐old mice of both genotypes (Figure 5d).

## 3 DISCUSSION

Here, we used a genetic mouse model of *G6PD* overexpression to show that increased NADPH production efficiently tempers the progression of early‐onset ARHL. Overall, our findings suggest that *G6PD* overexpression buffers ROS production and protects against oxidative damage in the cochlea, supporting the role of oxidative stress in age‐related cochlear degeneration and highlighting a potential new therapy based on the maintenance of NADPH levels.

The molecular mechanisms responsible for the age‐related basal–apical cellular degeneration of the cochlea are incompletely understood, but the expression of a distinct set of antioxidant enzymes along this axis might underscore the differential susceptibility of cochlear cell types to injury (Sha et al., 2001). ARHL is closely linked to increased levels of ROS (Martinez‐Vega et al., 2015; Wang & Puel, 2018), which participate in the onset and progression of the majority of age‐related diseases (Balaban et al., 2005). In this context, blocking the expression of detoxifying enzymes such as SOD1 expedites the onset of ARHL (McFadden et al., 1999), whereas overexpression of mitochondrial catalase reduces DNA oxidative damage and hair cell loss and prevents ARHL (Someya et al., 2009). Similarly, presbycusis is accelerated by micronutrient deficiencies that impair antioxidant defenses (Partearroyo et al., 2017). In the same line, inactivation of *Gpx1* increases noise‐induced damage in mice (Ohlemiller et al., 2000), whereas antioxidant gene therapy with catalase and SOD2 protects from aminoglycoside‐induced ototoxicity (Kawamoto et al., 2004). Accordingly, pharmacological antioxidant supplementation has proven to be efficacious in hearing protection against different stressors and during aging (Benkafadar et al., 2019; Marie et al., 2018; Muller & Barr‐Gillespie, 2015).

Using a gain‐of‐function model, we here demonstrate that moderate enhancement of *G6PD* expression in the cochlea has beneficial effects on redox metabolism and early‐onset ARHL progression. G6PD plays a key role in cellular redox metabolism by providing the NADPH needed for the activity of antioxidant enzymes and for protecting cells from oxidative stress‐induced apoptosis (Fico et al., 2004; Pandolfi et al., 1995). Accordingly, *G6PD*‐Tg mice show constitutively increased *G6PD* expression, elevated NADPH levels, and reduced levels of oxygen‐derived free radicals in brain and liver along aging, which results in an extended period of healthy life of the animals (Nobrega‐Pereira et al., 2016).

The transcription levels of mouse and human *G6PD* were greater in the cochleae of young *G6PD*‐Tg mice than in equivalent WT mice along the study. The expression of the genes coding for human and mouse G6PD decayed with age in both genotypes. Similarly, the expression levels of other major NADPH producing enzymes (*Pgd*, *Idh1*, and *Me1*) also declined with age in the cochlea. By contrast, G6PD activity did not decline with age, suggesting the existence of post‐transcriptional mechanisms that control G6PD levels and activity. Expression of the related genes *Pgd* and *Idh1* was also increased in 3‐month‐old *G6PD*‐Tg mice. The finding of increased *Idh1* transcription points to a tight regulation of cochlear cytosolic NADPH production by G6PD and IDH1. Indeed, IDH1 is the major source of NADPH in *G6pd* hypomorphic cochleae, maintaining cytosolic GSH levels and TRX activity, and therefore preserving auditory thresholds in these mice (White et al., 2017).

Higher levels of 6‐phosphogluconolactone due to elevated G6PD activity could explain the increase in *Pgd* expression to cope with the increased amount of substrate, as PGD catalyzes the second reaction in the pentose phosphate pathway (Stanton, 2012). However, PGD activity showed a more complex regulation and did not correlate with the transcriptional data. Increased NADPH consumption in young 3‐month‐old *G6PD*‐Tg mice may trigger *Idh1*, *Pgd*, and *G6pd* transcription, which are all involved in the NADPH metabolic network. Indeed, despite the increase in G6PD activity, there was a reduction in NADPH levels at this age. Also, 3‐month‐old *G6PD*‐Tg mice showed elevated levels of P22PHOX, a NOX activity upregulator, which decreased significantly at 9 months of age, mirroring the transgene expression profile and contrasting with NADPH levels.

Beyond the transcriptional induction of *G6PD* in young *G6PD*‐Tg mice, we detected a strong induction of major antioxidant enzymes. This orchestrated transcriptional induction might be driven by *Nrf2*, which is significantly upregulated in 3‐month‐old *G6PD*‐Tg cochleae and is a known positive regulator of *G6PD* expression (Tonelli et al., 2018). On the basis of our findings, we propose an intricate physiological mechanism in *G6PD*‐Tg cochlea where *Nrf2* transcription is secondarily induced in response to ROS production *via* NOX activation by NADPH overproduction. Concomitant with the age‐related decrease in the expression of *G6PD* coding genes and *Nrf2* there is a decrease in the expression of antioxidant enzymes. This might suggest a physiological adaptation to minimize the initial changes produced by *G6PD* overexpression, but still delaying hearing loss onset and progression, conferring upon *G6PD*‐Tg cochleae a more robust antioxidant capacity to suppress ROS production and oxidative damage. Hearing thresholds worsened at a slower rate in *G6PD*‐Tg mice than in age‐matched WT littermates, and the former showed better all‐frequency hearing at 9 months of age. Thus, the increased antioxidant defense mechanisms already operating in the 3‐month‐old transgenic cochleae cause less accumulation of oxidative damage between 3 and 9 months of age. At this adult age, the protective mechanisms at the molecular level are lost and the consequent cellular deterioration leads to the functional loss observed at the age of 12 months. Differences in the temporal patterns of aging observed between the cochleae and other *G6PD*‐Tg organs or tissues (Nobrega‐Pereira et al., 2016) might be explained by the high susceptibility to aging, early cellular degeneration and limited regeneration capacity of the cochlea.

The *G6PD*‐Tg mouse was generated on a C57BL/6 background, which is widely used to study ARHL because these mice develop premature hearing loss at the age of 6 months (Kane et al., 2012) with similar traits to human ARHL (Kusunoki et al., 2004). Specifically, similarities include the progressive high‐to‐low frequencies hearing loss, cell loss, and morphological alterations in the organ of Corti, SG, and lateral wall. The C57BL/6 strain harbors a mutation in Cadherin 23 gene (Cdh23ahl allele) that encodes for a defective form of this protein, one of the main components of the tip links connecting hair cell stereocilia. This mutation only impact cochlear aging and G6PD overexpression has a general anti‐aging effect, protecting other organs, including liver and the brain (Nobrega‐Pereira et al., 2016). Therefore, it is unlikely that the reported phenotype is related to the presence of the Cdh23 allele. We found that adult *G6PD*‐Tg mice had better hearing thresholds than WT littermates, which correlated with the preservation of sensory cells and SG neurons. Furthermore, adult *G6PD*‐Tg mice showed higher neuronal density and synaptic ribbons per IHC. By contrast, WT mice showed lower I–IV interpeak latencies than transgenic mice, which could indicate central auditory compensation of the peripheral delay (Fuentes‐Santamaria et al., 2016).

SG neurons are more vulnerable to oxidative stress than other cochlear cell populations (Miller, 2015). Accordingly, peroxynitrite‐derived modifications in proteins were significantly lower in *G6PD*‐Tg neurons than in WT counterparts. NOS enzymes require NADPH as a cofactor to catalyze NO formation and *iNos* induction, and an oxidative environment leads to the formation of peroxynitrite, a nitrating agent that damages DNA and proteins (Jiang et al., 2007). TRXs, GRXs, and peroxiredoxins prevent irreversible modification of protein residues, and their activity depends on the levels of NADPH and GSH (Hanschmann et al., 2013). *Txnrd1* and *Glrx1* are induced in *G6PD*‐Tg mice, potentially counteracting deleterious modifications in macromolecules. *Txnrd1* and *Txnrd2* genes encode for cytosolic and mitochondrial isoforms of TrxR, respectively. Reduced TrxR activity in the cytosol of 3‐month‐old *G6PD*‐Tg cochleae concurs with our hypothesis that G6PD overexpression delays hearing loss onset and progression. Indeed, activity is increased in adult *G6PD*‐Tg mice when the upregulation of the expression of antioxidant enzymes is lost and oxidative modifications in proteins are widespread, as observed with 3‐NT staining, and TrxR activity in *G6PD*‐Tg mice directly correlates with NADPH levels, suggesting that the overproduction of NADPH is utilized by NOXs and not TrxR enzymes in young G6PD‐Tg mice. NADPH is also an essential cofactor for oxidized glutathione (GSSG) reduction *via* glutathione reductase, therefore contributing to the maintenance of the GSH/GSSG ratio. An examination of the expression of genes involved in GSH synthesis and metabolism indicated that *Gss*, *Gclc*, and *Gclm* were unaltered in *G6PD*‐Tg cochleae, but *Ggt1 and Gsr* transcripts were upregulated. Higher levels of extracellular GSH could explain the transcriptional upregulation of *Ggt1* to recycle extra cytoplasmic GSH (Zhang et al., 2005). NADPH exhaustion due to NOX activation could explain the lower cytosolic GSR activity in young mice. On the other hand, mitochondrial GSR activity, which corresponds to the major isoform also encoded by the *Gsr* gene, was increased in young and adult *G6PD*‐Tg cochleae.

Preservation of mitochondrial function and energy supply is a key factor for aging progression. The PGC‐1 family of transcriptional coactivators and dependent genes are important for regulating mitochondrial gene expression to maintain an adequate number of mitochondria and reduce damage during aging (Wu et al., 1999). The cochleae of young *G6PD*‐Tg mice showed an increase in the expression of several canonical mitochondrial respiratory chain complex genes, which likely enhance *G6PD*‐Tg mitochondrial resistance to damage, as confirmed by complex IV staining in 9‐month‐old SG neurons. Furthermore, *Pgc1ß and Pparγ* upregulation in young *G6PD*‐Tg mice suggests that *G6PD* overexpression promotes mitochondrial gene expression. However, no differences were observed in *Pgc1α* or its downstream targets *Nrf1* and *Tfam*. As recently reported by (Kim et al., 2019), enhanced *Sirt1* expression could limit PARP1 activation by decreasing the NAD^+^ pool in the 3‐ and 9‐month‐old *G6PD*‐Tg cochleae.

In apparent contradiction with the aforementioned roles of G6PD, this enzyme has been reported to participate in the inflammatory response (Ham et al., 2013). Indeed, we found that elevated G6PD modulated the expression of pro‐inflammatory (*Il1ß* and *Tnfα)* and anti‐inflammatory (*Il10)* cytokines in the cochlea, but without an evident increase in inflammation as indicated by the expression of *FoxP3* and the absence of macrophage recruitment. Interestingly, the *G6PD*‐*Tg* cochlea maintains lower expression levels of *Il6* transcripts than the WT cochlea along the ages studied. IL6 has been linked to age‐associated chronic inflammation and ARHL (Watson et al., 2017).

Potentiation of the antioxidant defense by *G6PD* overexpression successfully protects from massive OHC death in adult *G6PD*‐Tg mice. Increased ROS levels from base to apex in the cochlea underlie the gradient of OHC susceptibility to damage and the characteristic basal–apical apoptotic death gradient (Sha et al., 2001). Despite the lower levels of cell death in the *G6PD*‐Tg cochlea, mice of both genotypes showed increased cochlear apoptosis between the ages of 3 and 9 months, as well as increased AKT activation to counteract apoptosis (Rajesh et al., 2015). MAPK signaling also participates in the cochlear response to oxidative stress during aging (Celaya et al., 2019), and *G6pd*‐deficient embryonic stem cells show increased MAPK phosphorylation and apoptosis after oxidative insult (Fico et al., 2004). Increased ROS promotes and sustains JNK activation and apoptosis (Kamata et al., 2005), and in the cochlea, the activation of stress‐activated kinases precedes apoptosis following cochlear insult and along aging (Sha et al., 2009). Accordingly, young *G6PD*‐Tg cochleae showed lower stress and apoptotic signaling than WT cochleae.

G6PD activity has been widely reported to be protective against oxidative stress *in vitro* and in animal models (Nobrega‐Pereira et al., 2016; Pandolfi et al., 1995; Salvemini et al., 1999). However, in neurodegenerative diseases, the efficaciousness of G6PD is controversial due to its dual role as antioxidant and a ROS generator (Tiwari, 2017). Our data indicate that in the cochlea, increased G6PD activity protects from oxidative damage.

In summary, we report here that the *G6PD*‐Tg mouse pro‐antioxidant state delays ARHL by counteracting age‐associated redox imbalance, oxidative‐induced damage, and mitochondrial dysfunction. Our findings indicate that G6PD is important to maintain cochlear redox balance and is a positive regulator of the inflammatory response. Our data further suggest that increasing NADPH levels is a potential strategy to prevent and ameliorate ARHL progression.

## 4 EXPERIMENTAL PROCEDURES

### 4.1 Mice

C57BL/6 (WT) (*n* = 37) and *G6PD*‐Tg (*n* = 34) mice containing the entire human *G6PD* gene including regulatory sequences were generated and genotyped as reported by Nobrega‐Pereira et al. (2016). Briefly, C57BL/6 and *G6PD*‐Tg mice were crossbred to generate litters of WT and transgenic mice. All animals used in this study were littermates. The C57BL/6 mouse strain is a classical model of ARHL due to a mutation in the *Cdh23* allele (Kane et al., 2012). No male–female differences were found between genotypes, and consequently, male and female mice were used for experiments. Animal procedures were in accordance with Spanish (RD 53/2013) and European legislation (Directive 2010/63/EU) and approved by the Spanish National Research Council (CSIC).

### 4.2 Hearing evaluation

ABR recordings were performed on a TDT system III evoked potential workstation (Tucker‐Davis Technologies, Alachua, FL, USA), as reported by Cediel et al. (2006). In brief, mice were anesthetized with ketamine (100 mg/kg; Imalgene 1000; Merial, Lyon, France) and xylazine (10 mg/kg; Rompun 2%; Bayer, Leverkusen, Germany) by intraperitoneal injection and the ABR tests were performed in a sound‐attenuating chamber. Two different sound stimuli, clicks and tone bursts, were generated with SigGenRP software (Tucker‐Davis Technologies). Stimuli were calibrated using SigCal software and an ACO Pacific 1⁄4‐inch microphone. Click stimuli were 0.1 ms and toneburst (4, 8, 16, 28, and 40 kHz) stimuli were 5‐ms duration (2.5 ms each for rise and decay, without plateau). The response was analyzed with BioSigRP software (Tucker‐Davis Technologies). Stainless steel needle electrodes were placed at the vertex and ventrolateral to the left and right ears for recording and a tweeter in open field configuration to deliver acoustic stimuli. Hearing thresholds were established at the lowest SPL level that produced a noticeable ABR five peaks wave and evoked a peak‐to‐peak voltage 2 SD above the mean background activity. Wave amplitudes, latencies, and inter‐wave latencies were determined at 70 dB SPL click stimulation. ABR measurements were performed in mice at 3 (*n* = 14 WT and *n* = 12 *G6PD*‐Tg), 6 (*n* = 4 WT and *n* = 4 *G6PD*‐Tg), 9 (*n* = 17 WT and *n* = 16 *G6PD*‐Tg), and 12 (*n* = 6 WT and *n* = 6 *G6PD*‐Tg) months of age.

### 4.3 Gene expression analysis

Inner ears were dissected after mice euthanasia with a sodium pentobarbital overdose (Vetoquinol, Spain) and immediately immersed in RNA*later* ^®^ solution (Sigma‐Aldrich, St. Louis, MO, USA). Adjacent tissues were carefully detached from the inner ear to avoid contamination. Finally, cochleae were isolated from vestibules and frozen in RNA*later* ^®^ solution. Cochlear RNA extraction from pooled cochlea (*n* = 3 mice per experimental group), quality determination, and cDNA generation were performed as reported by Celaya et al. (2019). Quantitative amplification was performed in triplicate on an Applied Biosystems 7900HT Real‐Time PCR System using either commercial TaqMan probes or gene‐specific primers (Tables 1 and 2). Data were collected after each amplification step and analyzed with SDS 2.2.2 software (Applied Biosystems, Foster City, CA, USA). The *18s gene* was used as a housekeeping gene and the n‐fold differences were calculated using the 2^–ΔΔCt^ method. Total *G6PD* expression levels are represented as the sum of the 2^–ΔCt^ for the murine and human primers.

**Table 1:** Primers designed for real‐time RT‐qPCR experiments with SYBR Green

Gene	Forward primer (5′−3′)	Reverse primer (5′−3′)
*G6PD*	GGAGGGCGACGACGACGAAG	TCGGGCAGAAGGCCATCCCG
*G6pd*	CCGGAAACTGGCTGTGCGCT	CCAGGTCACCCGATGCACCC
*Ggt1*	CTCGGTGACCCAAAGTTTGTC	GCGTAGAACTCAGAGCTCATGTTG
*Glrx1*	CCAGTGCGATTCAAGATTATTTACA	GCCTATGCAGTCTTTACCTATGAAGA
*Glrx2*	TGTGAACCAGATCCAAGAAACAAT	GGAACAGTAAGAGCAGGATGTTTTT
*Ho1*	GAGGCTAAGACCGCCTTCCT	TTGTGTTCCTCTGTCAGCATCAC
*Idh1*	CATGTACCAGAAAGGGCAAGAGA	CCTCGGGACCAGGCAAA
*Me1*	TGACCAAGGGACGTGCAA	GGGAGAGTGACTGGATCAAAAGG
*Mpo*	CATGCCCACCGAATGACA	GCTTCGTCTGTTGTTGCAGTGT
*nNos*	CCGCCAAAACCTGCAAAG	TGTGGAGACGCACGAAGATG
*Nrf2*	GCCTTGTACTTTGAAGACTGTATGCA	AAGCGACTCATGGTCATCTACAAAT
*Pgd*	AGAGGCTTGGCCCCACAT	CCGGTTCCCACTTTTGCA
*Prdx6*	GACGCTAACAACATGCCTGTGA	CAGTTTCTTGTCAGGGCCAAA
*Sod2*	GGCCAAGGGAGATGTTACAA	GCTTGATAGCCTCCAGCAAC
*Txnrd1*	CAGCGAGGAGACCATAGAGG	GCACATTGGTCTGCTCTTCA
*Txnrd2*	GCTTCTGGCAAGGAAGACAC	CCCTCAGCAACATCTCCAAT
*Ndufs1*	AATGAAGGGCTGGAATATCTTGAC	CATCACACCTTCTCTGGCTTTCT
*Sdha*	GGTGTTGCTGTGTGGCTGAT	CATATCGCAGAGATCTTCCATACAA
*Uqcrc2*	ACCCGTGGGATTGAAGCA	CGCCATGTTTTCCCTTGTTG
*Mtco1*	GCTAGCCGCAGGCATTACTATAC	GCGGGATCAAAGAAAGTTGTG
*Mtatp6*	CACTATGAGCTGGAGCCGTAATT	GAAGTGGGCAAGTGAGCTTTTT
*Pgc1α*	ACCCACAGGATCAGAACAAACC	TCTTCGCTTTATTGCTCCATGA
*Pgc1β*	CGGAAGAACTTCAGACGTGAGA	CATGTCACTGGAGAGATTTCGAAT
*Pprc1*	AGAAGGAGCGTGCAATAGAAGAG	TTCAGCTCCGACCGAGTCAT
*Pparα*	CCCTGTTTGTGGCTGCTATAATT	GCAACTTCTCAATGTAGCCTATGTTT
*Pparγ*	CCCAATGGTTGCTGATTACAAA	AATAATAAGGTGGAGATGCAGGTTCT
*Nrf1*	TTGGCGCAGCACCTTTG	GGTCTTCCAGGATCATGCTCTT
*Tfam*	TCCAGGAGGCAAAGGATGAT	TCCTCAGGAGACAGATTTTTCCA
*Tfb1m*	CCGAGGGCTTGGAATGTTATT	CAGCCTTCCAGTGCTTTCG
*Tfb2m*	GGTCGTCATTTAGTGTGCATACG	CTGATTCCCCGTGCTTTGA
*Alas1*	AGGAAAGAGGCTGCTCAAAGC	ATCCTCTCCATCGGTTTTCACA
*Ucp2*	GCCTCTGGAAAGGGACTTCTC	ACCAGCTCAGCACAGTTGACA
*Sirt1*	CGGCTACCGAGGTCCATATACTT	TTCGAGGATCGGTGCCAAT
*Sirt3*	CGGCTCTATACACAGAACATCGA	GTGGGCTTCAACCAGCTTTG

Primers were designed using Primer Express 3.0 software and the mouse gene sequences available on the Ensembl genome database.

**Table 2:** TaqMan probes used in real‐time RT‐qPCR experiments

Symbol	Gene	Reference
*Cat*	*Catalase*	Mm00437992_m1
*FoxP3*	*Forkhead box P3*	Mm00475162_m1
*Gclc*	*Glutamate‐cysteine ligase, catalytic subunit*	Mm00802655_m1
*Gclm*	*Glutamate‐cysteine ligase, modifier subunit*	Mm00514996_m1
*Gpx1*	*Glutathione peroxidase 1*	Mm00656767_g1
*Gpx4*	*Glutathione peroxidase 4*	Mm00515041_m1
*Gsr*	*Glutathione reductase*	Mm00439154_m1
*Gss*	*Glutathione synthetase*	Mm00515065_m1
*Il1β*	*Interleukin 1 beta*	Mm00434228_m1
*Il6*	*Interleukin 6*	Mm00446190_m1
*iNos*	Inducible nitric oxide synthase	Mm00440502_m1
*P22phox*	*Cytochrome b‐245, Cyba*	Mm00514478_m1
*Slc26a5*	*Prestin*	Mm00446145_m1
*Tgfβ1*	*Transforming growth factor, beta 1*	Mm01178820_m1
*Tnfα*	*Transforming nuclear factor*	Mm99999068_m1
*Ucp1*	*Uncoupling protein 1*	Mm01244861_m1

### 4.4 Cochlear morphology, immunohistochemistry, and immunohistofluorescence

For histological analysis, 3‐ and 9‐month‐old mice (*n* = 4 per experimental group) were perfused with 4% paraformaldehyde (PFA; Merck, Darmstadt, Germany) in PBS. Dissected inner ears were post‐fixed with 4% PFA, decalcified in 5% EDTA (Sigma‐Aldrich), and embedded in paraffin. Paraffin cochlear midmodiolar sections (5 μm) were obtained on a RM2155 microtome (Leica Microsystems, Deerfield, IL, USA) and were either hematoxylin–eosin‐stained or immunostained with chicken anti‐myelin P0 (MyeP0, 1:100, CH23009; Neuromics, Edina, MN, USA) or rabbit anti‐3‐nitrotyrosine (3‐NT, 1:200, AB5411; Millipore, Billerica, MA, USA) antibodies. Images were acquired with a Zeiss AxioPhot microscope (Carl Zeiss, Jena, Germany) and captured with an Olympus DP70 digital camera (Melville, NY, USA). MyeP0 and 3‐NT staining intensity was quantified delimiting the same region of interest (ROI) in the SG of each sample. Four consecutive serial sections per sample were studied and analyzed using Fiji software (Schindelin et al., 2012). For immunohistofluorescence, frozen cryostat cross sections (10 μm) obtained on a Cryocut 1950 (Leica Microsystems) from Tissue‐Tek‐embedded inner ears were incubated with goat anti‐IBA1 (1:100, Ab5076; Abcam, Cambridge, UK) or mouse anti‐cytochrome c oxidase subunit I (1:100; Molecular Probes, Eugene, OR, USA). Fluorescent images were taken with an epifluorescence (Nikon 90i, Tokyo, Japan) or a confocal laser‐scanning microscope (Zeiss LSM710, Carl Zeiss). Total IBA1 intensity and mean cytochrome c oxidase subunit I intensity in the spiral ligament and SG, respectively, were calculated with Fiji software (Schindelin et al., 2012) for each cochlear turn in 4 serial cryosections per animal prepared from at least three mice of each genotype and age group, as reported by Celaya et al. (2019).

### 4.5 Organ of Corti whole mount immunohistofluorescence, cochleogram plotting, hair cell, and synapsis quantification

PFA‐perfused, post‐fixed and decalcified cochleae from 9‐month‐old mice were dissected into 5 half‐turns from apex to base. Organs of Corti were permeabilized with 1% Triton X‐100 (Merck), blocked with 5% normal goat serum (Sigma‐Aldrich), and incubated overnight at 4°C with the hair cell marker rabbit anti‐Myo7a (1:150, PT‐25‐6790; Proteus, Ramona, CA, USA) and nerve fiber marker mouse anti‐neurofilament (1:100, CBL212; Merck), or for 19 h at 37°C with mouse anti‐CtBP2 (1:200, 612044) and mouse anti‐GluR2/3 (1:1000, MAB397, Millipore). Sections were then incubated with the corresponding Alexa Fluor secondary antibodies and Alexa Fluor 647 Phalloidin (1:1000, A22287; Thermo Fisher Scientific, Waltham, MA, USA) at 1:200 for 2 h at room temperature. The half‐turns were finally incubated with DAPI (1:1000; Thermo Fisher Scientific) and mounted with Prolong reagent (Thermo Fisher Scientific), and low magnification fluorescent images were taken with a Nikon 90i microscope. These images were used for cochleogram plotting using a custom Fiji plugin, as reported by Liberman et al. (2014), and for immunofluorescence quantification with Fiji software as reported by Schindelin et al. (2012). IHC and OHC numbers were counted in 200 μm of the basilar membrane in the 8 (apical), 16–20 (middle), and 32–40 (basal) kHz regions located 15%–20%, 35%–40%, and 65%–70%, respectively, from the apex. Neurofilament staining intensity and the number of neural fibers crossing the tunnel of Corti were measured in parallel (*n* = 3 mice per group). Co‐localized presynaptic ribbons and postsynaptic glutamate receptor patches were counted from each confocal *z*‐stack using IMARIS software (Bitplane Inc., Saint Paul, MN, USA) (*n* = 3 mice per group). A spot of each signal (CtBP2 and GluR273) was created in independent channels using an XY diameter of 0.5 μm for CtBP2 and 0.7 μm for GluR2/3, a corrected PSF Z diameter of 2 μm, background subtraction function, and manually adjusting the intensity criterion to capture all elements of interest. Spot co‐localization measurements were used to analyze pairing of presynaptic and postsynaptic elements. A threshold was set at 0.75 μm to define the juxtaposition of two different puncta. The computed results were corroborated by visual inspection of the puncta. Representative fluorescent stack images were acquired with a Zeiss LSM710 confocal laser‐scanning microscope (Carl Zeiss) at the specified cochlear regions with a glycerol‐immersion objective (63×).

### 4.6 TUNEL assay

Post‐fixed and decalcified cochleae from 9‐month‐old mice (*n* = 3 per group) were dissected into 5 half‐turns from apex to base. Apoptosis was evaluated by TdT‐mediated dUTP nick‐end labeling (TUNEL) (Dead‐End Fluorometric TUNEL System, Promega, Madison, WI, USA). Organ of Corti half‐turns were post‐fixed using 4% PFA pH 7.4, permeabilized with 0.2% Triton X‐100 (Merck), and incubated with the TdT enzyme for 1 h at 37°C. The half‐turns were then incubated with DAPI (1:1000) and mounted with Prolong reagent (both from Thermo Fisher Scientific) before visualizing on a Zeiss LSM710 laser‐scanning confocal microscope (Carl Zeiss). TUNEL‐positive OHCs were counted in 200 μm of basilar membrane and scored as a percentage of the total number of OHC.

### 4.7 Protein extraction and immunoblotting

Whole cochleae protein extracts were prepared from 3 mice as described (Sanchez‐Calderon et al., 2010). An equal volume of extracts was resolved using SDS‐PAGE, followed by transfer to PVDF membranes (0.2 μm; Bio‐Rad Laboratories, Hercules, CA, USA) using the Bio‐Rad Trans Blot TURBO apparatus. Membranes were blocked with 5% BSA or non‐fat dried milk in 0.075% Tween, 1 mM TBS, and incubated overnight with the following antibodies: rabbit anti‐P‐p38 (1:1000, 9211), rabbit anti‐P‐JNK (1:1000, 4668), rabbit anti‐P‐ERK (1:1000, 9101) (all from Cell Signaling Technology, Danvers, MA, USA), rabbit anti‐p22phox (1:250, sc‐20781; Santa Cruz Biotechnology, Dallas, TX, USA), rabbit anti‐BAX (Nt) (1:000, ABC11; Merck), rabbit anti‐PARP1 (1:1000, Sc7150; Santa Cruz Biotechnology), or rabbit anti‐PI3 K (1:10,000, in‐house). Membranes were then incubated with a peroxidase‐conjugated secondary antibody for 1 h at room temperature, and bands were visualized using Clarity™ Western ECL Substrate (Bio‐Rad) using an ImageQuant LAS4000 mini digital camera (GE Healthcare Bio‐Sciences, Pittsburgh, PA, USA) and densities were quantified using Image Quant TL software.

### 4.8 Isolation of mitochondrial, cytosolic fractions, enzymatic activity, and NADPH measurements

Mitochondrial and cytosolic fractions were isolated as described by White et al. (2018) with some modifications to the protocol. Pooled cochlea (*n* = 3 mice per experimental group) were homogenized in 450 µl of buffer containing 10 mM Tris, 1 mM EDTA, 320 mM sucrose, pH 7.4, on ice using a Dounce Tissue Grinder (Wheaton, Millville, NJ, USA). DNA was then sheared using a syringe with a 25‐G needle and centrifuged at 850 *g* at 4°C for 5 min to obtain the nuclear fraction (pellet). The supernatant was then centrifuged at 16,000 *g* at 4°C for 10 min to obtain the cytosolic fraction (supernatant). Finally, the mitochondrial fraction was obtained resuspending the pellet in 200 µl 1% NP‐40 buffer and centrifuging at 14,000 *g* for 10 min. Protein concentrations were determined using the DC Protein Assay kit (Bio‐Rad Laboratories). G6PD and PGD activities were measured in cytosolic fractions as reported by White et al. (2017). In brief, 20 μl of sample was mixed with 180 μl of 50 mM Tris 1 mM MgCl, pH 8.1. Total (G6PD + PGD) activity was measured by the addition of glucose‐6‐phosphate (0.2 mM) and 6‐phosphogluconate (0.2 mM), whereas PGD activity was measured by adding only 6‐phosphogluconate (0.2 mM). The reaction was started by adding NADP^+^ (0.1 mM) in all wells. Reaction rate was measured at 340 nm every 20 s for 10 min. G6PD activity was obtained by subtracting PGD activity from total (G6PD + PGD) activity. GSR and TrxR activities were measured using the Glutathione Reductase Assay Kit (Sigma‐Aldrich, GRSA‐1 KT) and the Thioredoxin Reductase Assay Kit (Abcam, ab83463), respectively, following the manufacturers’ instructions in cytosolic and mitochondrial fractions. NADPH levels were determined using the NADP/NADPH Assay Kit (Abcam, ab65349) according to the manufacturer's instructions in pooled inner ears (*n* = 4 mice per experimental group) homogenized in 600 µl of the extraction buffer supplied with the kit. All spectrophotometric measurements were performed at least in duplicates in a 96‐well format in a VERSA max Tunable Microplate Reader (Molecular Devices, CA, USA).

### 4.9 Statistical analysis

Data analysis was performed by two‐tailed Student's *t* test after a Levene's or Fisher's test of equality of variances with SPSS v25.0 (IBM Corp., Armonk, NY, USA) or Microsoft Excel software (Microsoft Corp., Seattle, WA, USA). Data are expressed as mean ± SEM. Results were considered significant at *p* < 0.05.

## CONFLICT OF INTEREST

The authors declare no conflict of interest.

## AUTHOR CONTRIBUTIONS

JMBM contributed to conception and design, acquisition of data, data curation, formal analysis, supervision, investigation, visualization, and writing of original draft; AMC contributed to acquisition of data, data curation, formal analysis, supervision, investigation writing of review, and editing; SHP contributed to acquisition of data, data curation, writing of review, and editing; JW contributed to resources, writing of review, and editing; MS contributed to conception and design and provision of resources; and IVN contributed to conception and design, formal analysis, supervision, visualization, writing of original draft, funding acquisition, and project administration.

## Supporting information

Fig S1

Fig S2

Fig S3

## Data Availability

The data that support the findings of this study are available on request from the corresponding author.
