# Supplementary figures and images for: G6PD overexpression protects from oxidative stress and age‐related hearing loss
Source: Aging Cell. 2020 Nov 22;19(12):e13275. doi: 10.1111/acel.13275 (PMC7744953; doi:10.1111/acel.13275)

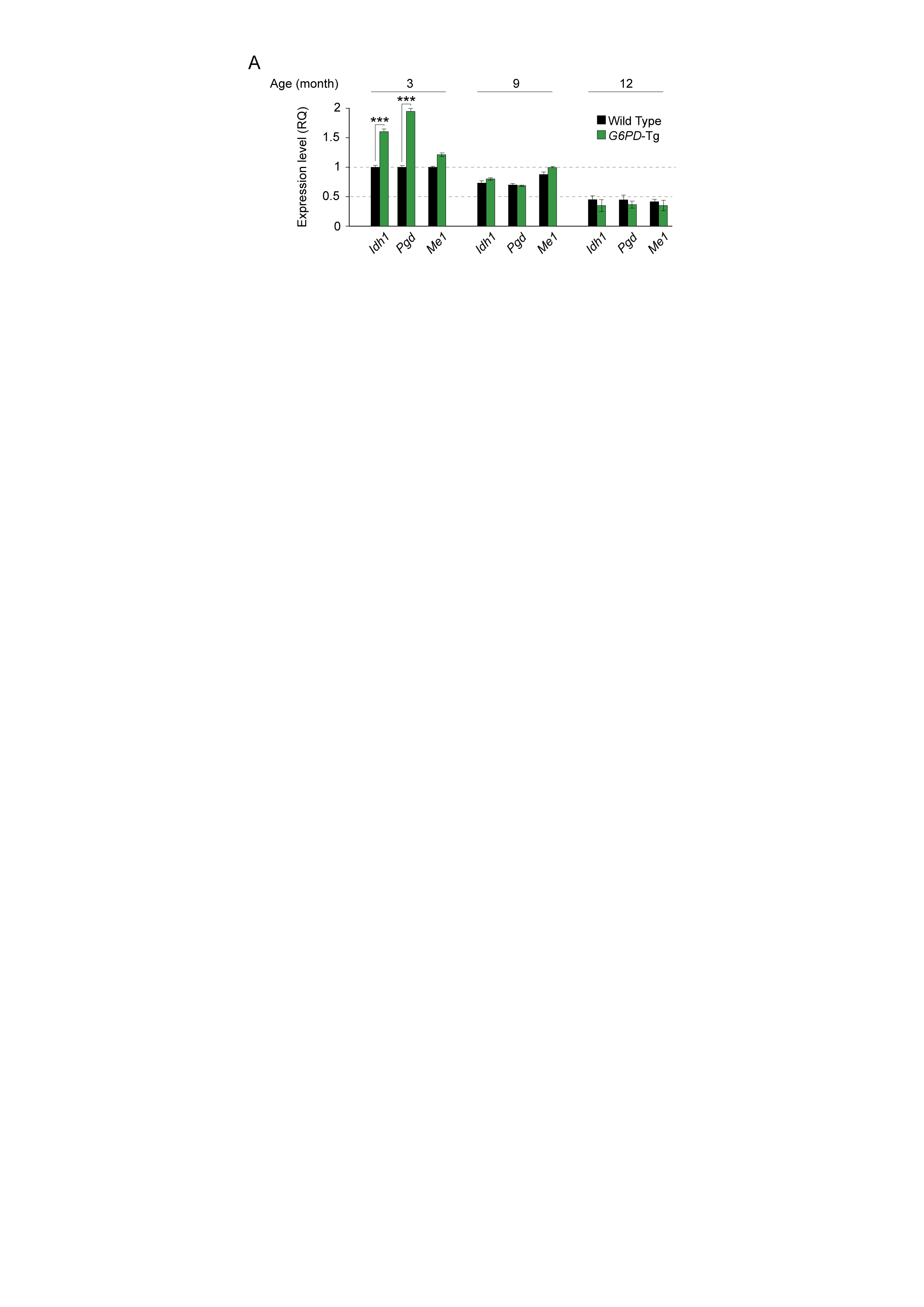

Supplement: Supplementary file 1 — Fig S1 [file ACEL-19-e13275-s001.tif]

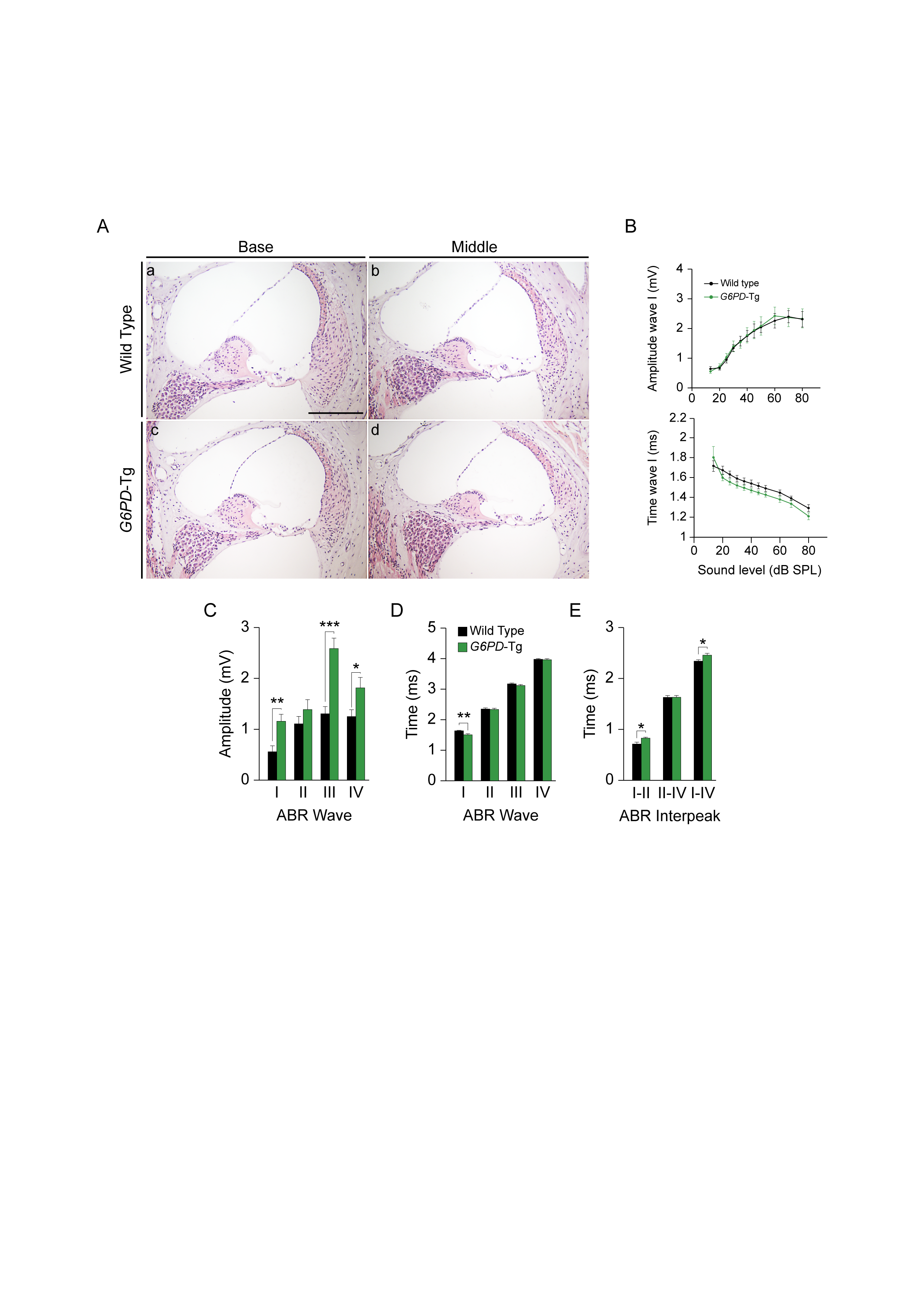

Supplement: Supplementary file 2 — Fig S2 [file ACEL-19-e13275-s002.tif]

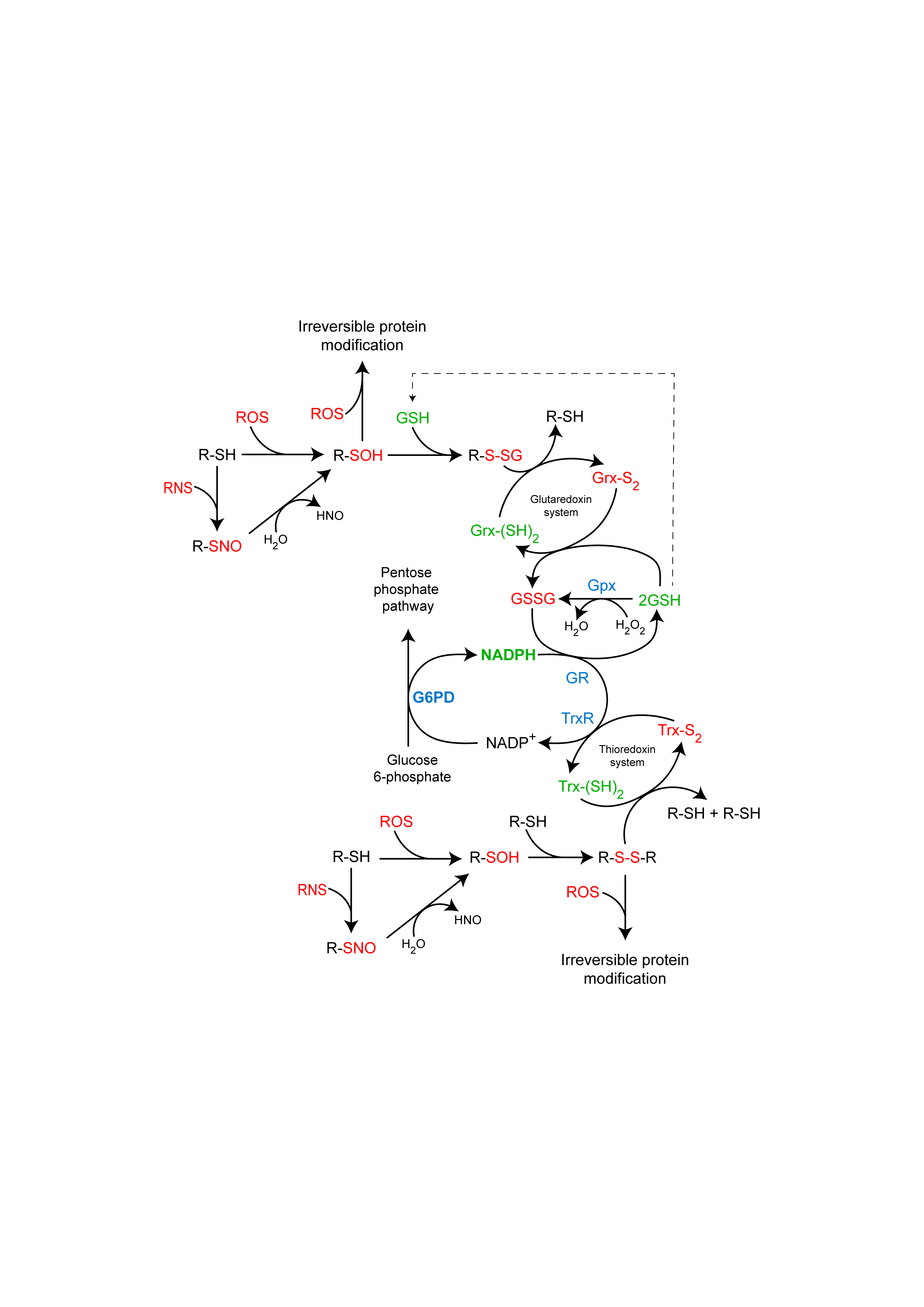

Supplement: Supplementary file 3 — Fig S3 [file ACEL-19-e13275-s003.tif]
